# High-Resolution MR for Follow-Up of Intracranial Steno-Occlusive Disease Treated by Endovascular Treatment

**DOI:** 10.3389/fneur.2021.706645

**Published:** 2021-12-24

**Authors:** Junjie Wang, Shun Zhang, Jun Lu, Peng Qi, Shen Hu, Ximeng Yang, Kunpeng Chen, Daming Wang

**Affiliations:** ^1^Department of Neurosurgery, Beijing Hospital, National Center of Gerontology, Institute of Geriatric Medicine, Chinese Academy of Medical Sciences, Beijing, China; ^2^Graduate School of Peking Union Medical College, Beijing, China

**Keywords:** HR-MRI (high-resolution MRI), ICAD (intracranial artery disease), DSA (digital subtraction angiography), follow-up, endovascular recanalization

## Abstract

**Background and Purpose:** An endovascular recanalization is an alternative option for symptomatic intracranial atherosclerotic steno-occlusive disease (ICAD). Accurate non-invasive alternatives to digital subtraction angiography (DSA) for follow-up imaging after endovascular treatment are desirable. We aimed to evaluate the image quality and diagnostic performance of high-resolution magnetic imaging in follow-up using DSA as a reference.

**Materials and Methods:** From January 2017 to June 2021, data from 35 patients with 40 intracranial steno-occlusive lesions who underwent endovascular recanalization and received high-resolution magnetic resonance (HR-MR) follow-up were retrospectively collected in our prospective database. Studies were evaluated for the quality of visualization of the vessel lumen, restenosis rate, and accuracy of high-resolution magnetic resonance (HR-MR) with DSA used as the reference standard. Intraclass correlation coefficient (ICC) analyses were performed to assess the agreement between the two different readers.

**Results:** In total, 40 intracranial steno-occlusive lesions in 35 patients, with 34 lesions undergoing balloon angioplasty [including 16 drug-coated balloons (DCBs)] and 8 lesions undergoing stenting were enrolled. The median age was 63.6 years (IQR 58.5–70.0 years), and the mean imaging follow-up time was 9.5 months (IQR 4.8–12.5 months). The median degrees of preprocedural and residual stenosis were 85.0% (IQR 75.0–99.0%) and 32.8% (IQR 15.0–50.0%), respectively. Intracranial periprocedural complications occurred in 1 (3.6%) patient. In the case of a stainless-steel stent (*n* = 1), there was a signal drop at the level of the vessel, which did not allow evaluation of the vessel lumen. However, this was visible in the case of nitinol stents (*n* = 7) and angioplasty (*n* = 34). The overall restenosis rate was 25.8% (*n* = 9). The DCB subgroup showed a lower rate of restenosis than the percutaneous transluminal angioplasty (PTA) subgroup [5.3% (2/13) vs. 35.7% (5/14)].

**Conclusion:** High-resolution magnetic resonance may be a reliable non-invasive method for demonstrating the vessel lumen and diagnostic follow-up after endovascular recanalization for ICAD. Compared with MR angiography (MRA), HR-MR showed a higher inter-reader agreement and could provide more information after endovascular recanalization, such as enhancement of the vessel wall.

## Introduction

Intracranial artery disease (ICAD) is one of the major causes of ischemic stroke and neurologic symptoms, especially in Asians (1–4). Endovascular treatment (EVT) has been an alternative option for patients refractory to medication, as it can restore blood flow and shows promise to prevent the recurrence of stroke. However, EVT for ICAD is associated with a high-restenosis rate (up to 30%), which accounts for most subsequent recurrence of ischemic events (5).

Lumenography, such as digital subtraction angiography (DSA), CT angiography (CTA), and MR angiography (MRA), has been essential and widely used in the diagnosis of ICAD and follow-up imaging (6). Among these methods, DSA is thought to be the standard criterion tool because of its superior spatial and temporal resolution (7, 8). However, it is also an invasive procedure with the risk of neurologic complications and radiation exposure (9, 10). Conventional MRA and CTA can be used as minimally invasive methods to assess intracranial stenosis, but artifacts can be a problem in the luminal evaluation compared with DSA. Accurate non-invasive alternatives to DSA for follow-up imaging after EVT are desirable.

High-resolution magnetic resonance (HR-MR) imaging has been recently introduced as a minimally invasive-advanced imaging technique for directly identifying the intracranial arterial wall (11, 12), which may correlate with luminal angiography (13–15). In the recent years, some studies have shown the feasibility of HR-MR in the evaluation and characterization of intracranial atherosclerosis and steno-occlusive lesions (12, 16, 17). However, data on follow-up after EVT based on HR-MR are limited. Therefore, we report our initial experience using HR-MR for evaluating angiographic follow-up outcomes of ICAD after EVT.

This retrospective study was approved by our institutional review board and information of the patient was anonymized and reidentified before the assessment.

## Methods

### Patient Selection

The studies involving human participants were reviewed and approved by the institutional ethics committee at Beijing Hospital. The patients provided written informed consent to participate in this study. Prior to the intervention, patients gave their informed consent to the operation. Patients (≥18 years) with symptomatic, intracranial high-grade stenosis (WASID ≥ 70%) or occlusion and elective [ipsilateral hypoperfusion (≥40% decrease in cerebral blood flow in the territory distal to the target lesion in CT perfusion)] endovascular recanalization between January 2017 and June 2020 who underwent HR-MR follow-up were included in this retrospective data analysis. Patients with hyperacute (0–24 h) stroke or with asymptomatic steno-occlusive lesions and without follow-up were excluded.

### Data Collection and Follow-Up Outcomes

Demographical, clinical, angiographical, and periprocedural data were collected. The primary follow-up outcomes were angiographic restenosis, ISR, and recurrent ischemic events. Restenosis or ISR was defined as >50% stenosis within or immediately adjacent (within 5 mm) to the treated segment and >20% absolute luminal loss. Symptomatic restenosis was defined as restenosis associated with ischemic symptoms of the offending vessel territory.

### Strategy of Endovascular Treatment

Digital subtraction angiography was performed for all the patients and the strategy of endovascular treatment was decided according to the site and characteristics of the target lesions and based on the experience and preference of the operators. In general, drug-coated balloons (DCBs) (SeQuent Please, Braun Medical, Melsungen, Germany) with balloon predilatation were selected in lesions with tortuous arterial access or with a significant mismatch in the diameter between the proximal and distal segments or lesions in vessels with small reference diameters. Balloon dilation (BD) alone (Tazuna, Terumo, Japan; Ryujin, Terumo, Japan; Gateway, Stryker Neurovascular, USA; Sprinter, Medtronic, USA) was performed in patients with more tortuous arterial access for which DCB or stenting was considered improper by operators. Stenting (Wingspan, Stryker Neurovascular, USA; Solitaire, Medtronic, USA; Apollo, MicroPort Medical, China) was preferred in lesions with straightforward arterial access or was deployed for the treatment of dissection after balloon dilation.

### Procedure and Periprocedural Management

Endovascular procedures were performed by experienced neurointerventionists with the patients under general anesthesia. Prior to the intervention, all the patients were under dual antiplatelet therapy (DATP) with aspirin and clopidogrel. An intravenous heparin regimen was administered to maintain an activated clotting time between 250 and 300 s during the procedure. A 6F-guiding catheter was introduced through the common femoral artery and guided into the ICA or VA proximal to the target lesion. The precise length and diameter of the lesion were assessed by three-dimensional (3D) DSA prior to the recanalization procedure. Under the fluoroscopic guidance, a microwire was first passed through the intracranial steno-occlusive lesion. Then, one of the three kinds of interventional procedures (DCB, BD, and stenting) was performed based on the experience of the operators. The choice of strategy is as described earlier.

Dual antiplatelet therapy was maintained for at least 6 months and aspirin or clopidogrel alone was continued daily afterward. Long-term management of individual medical risk factors, such as blood pressure, cholesterol, and diabetes mellitus, was implemented.

### Imaging Protocol

High-resolution magnetic resonance was performed with a 3.0-T MR scanner (Achieva; Philips Health care, Best, The Netherlands) with a 16-channel NV coil. The HR-MRI sequences included 3D time of flight MRA and pre and postcontrast T1W imaging (VISTA). The parameters were as follows for time-of-flight MRA: repetition time = 25 ms; echo time = 3.45 ms; field-of-view = 180 mm × 180 mm; and acquired resolution = 0.55 mm × 0.55 mm × 1.1 mm. For T1W imaging, the parameters were: repetition time = 800 ms; echo time = 18 ms; field of view = 200 mm × 180 mm × 40 mm; and acquired resolution = 0.6 mm × 0.6 mm × 0.6 mm. Gadoteric acid meglumine (Dotarem; Guerbet, Aulnay-sous-Bois, France) was intravenously injected (0.1 mmol/kg of bodyweight). T1W imaging was repeated 5 min after injection.

Digital subtraction angiography was performed in 4 vessels using a biplane system, including high-resolution 3D rotational angiography (FD20; Philips Health care, Best, The Netherlands). Transfemoral access was used, and selective injection of a contrast medium, either iohexol (350 mg of iodine/ml; Beijing Beilu Pharmaceutical Corporation, China) or iodixanol (320 mg of iodine/ml, Visipaque; GE Healthcare, USA), was performed at a rate of 4 ml (for internal carotid artery) or 3 ml (for vertebral artery) per second using an injector (Liebel-Flarsheim Angiomat Illumina, USA). The parameters were as follows: matrix, 1,024; FOV, 310 mm.

### Imaging Analysis

Two neuroradiologists independently assessed images on the basis of the PACS workstation (Neusoft, Shenyang, China; version 5.5.0) for MR images and the interventional workspot (Philips Medical Systems, Best, The Netherlands; Version 1.4.1) for 3D rotational DSA data.

The image quality of HR-MR was first assessed by an experienced reviewer using a 3-point scale, where 1 = poor [low signal-to-noise (SNR) and obscured vessel wall or lumen boundaries], 2 = fair (passable SNR with a few motions or blood artifacts, distinguishable vessel wall, but partially obscured vessel lumen and wall boundaries), and 3 = good (high SNR without artifacts, clearly displaying vessel lumen boundary and wall). Images with an image quality of 1 were excluded from further analyses.

**Figure 1:**
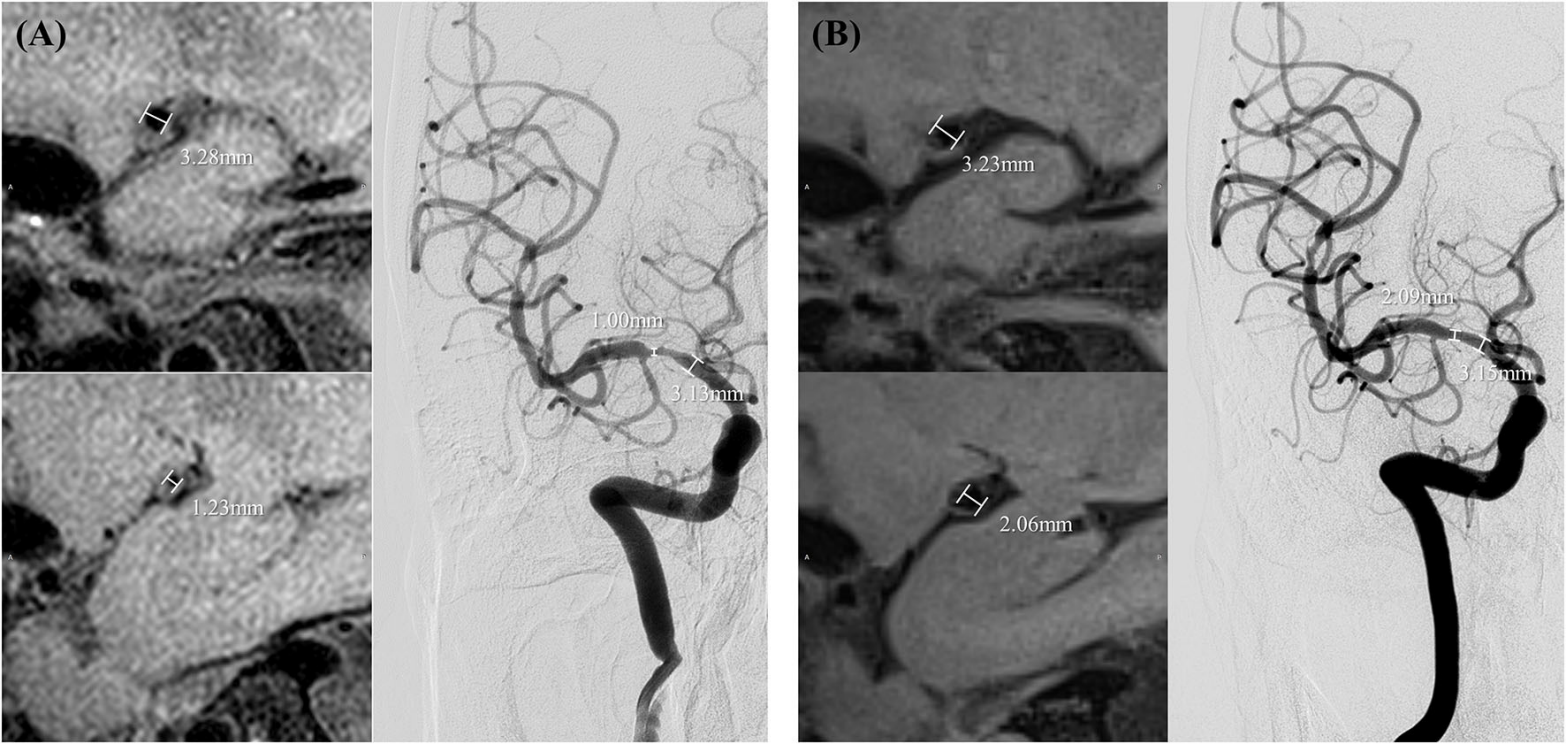
Measurements of the degree of stenosis and minimal luminal diameter in preprocedural high-resolution magnetic resonance (HR-MR) and digital subtraction angiography (DSA) **(A)** and follow-up HR-MR and DSA **(B)**.

The following parameters were measured: (1) the arterial luminal diameter (i.e., stenosis diameter and reference diameter). MR data were measured manually in 0.6-mm-thick cross-sections reformatted from HR-MR and 0.6-mm-thick cross-sections from 3D tetralogy of fallot (TOF). Data of DSA were collected from 3D reconstruction of images (when 3D images were not available, data were measured manually in 2-dimensional DSA images); (2) stenosis degree (%) calculated according to the Warfarin–Aspirin Symptomatic Intracranial Disease trial criteria; (3) presence and change of wall enhancement at the site of steno-occlusive lesions in HR-MR. According to the enhancement in the follow-up images compared with the preprocedural images, the images were evaluated as: increase, little change, or less (Figure 1).

### Statistical Analysis

Continuous variables are presented as the mean ± SD and categorical variables are reported as the frequencies (percentages), as appropriate. For continuous variables, the agreement between HR-MR and DSA was assessed by using the intraclass correlation coefficient (ICC) and the Bland–Altman analyses. The level of agreement was categorized as follows: poor (ICC = 0–0.20), fair (ICC = 0.21–0.40), moderate (ICC = 0.41–0.60), good (ICC = 0.61–0.80), and excellent (ICC = 0.81–1.00). Differences between proportions were assessed by chi-squared analysis. All the statistical analyses were performed using R Statistical Software version 3.6.2 (Foundation for Statistical Computing, Vienna, Austria). *p* <0.05 was considered to indicate statistically significant.

## Results

### Patient Characteristics

Overall, 40 intracranial steno-occlusive lesions in 35 patients (22 males, 13 females; median age, 63.6 years, IQR 58.5–70.0 years), with 32 lesions undergoing balloon angioplasty (including 16 DCB) and 8 lesions undergoing stenting, were identified. Most treated lesions were located in the anterior circulation. The median preprocedural and residual degrees of stenosis were 85.0% (IQR 75.0–99.0%) and 32.8% (IQR 15.0–50.0%), respectively. In one patient, dissection of the M2 segment of the MCA with consecutive subarachnoid hemorrhage occurred during the interventional maneuver. However, the patient recovered completely from this incident. The median radiological follow-up was 9.5 months (IQR 4.8–12.5 months, range 1.8–29.0 months).

On follow-up, the overall restenosis rate was 25.8% (*n* = 9). Of these 9 patients with restenosis, 2 patients were in the stent group (25.0%, 2/8). The other 7 cases were in the angioplasty group (21.9%, 7/32). One patient in the stent group suffered from symptomatic restenosis and he received further revascularization with the insertion of another stent in the intracranial vertebral artery. The other 8 patients were asymptomatic and received medical treatment. The DCB subgroup showed a lower rate of restenosis than the percutaneous transluminal angioplasty (PTA) subgroup [12.5 (2/16) vs. 31.2% (5/16)] (Table 1).

**Table 1:** Characteristics of patients who underwent endovascular recanalization and received high-resolution magnetic resonance (HR-MR) follow-up.

**Characteristics**	**Overall**	**DCB**	**PTA**	**Stenting**
Age (years)	63.6(58.5-70.0)	62.7(58.8-69.5)	61.8(57.0-67.0)	69.8(68.0-75.5)
Gender (male)	62.9% (22/35)	56.3% (9/16)	61.5% (8/13)	83.3% (5/6)
Radiological follow-up time (months)	9.5(4.8–12.5)	9.5(6.0–12.0)	7.6(2.8–11.0)	13.5(5.8–17.8)
Pre-procedural degrees of stenosis	85% (75.0–99.0%)	80.0% (70.0–86.3%)	91.3% (85.0–99.3%)	81.8% (73.8–92.3%)
Residual degree of stenosis	32.8% (15.0–50.0%)	29.0% (15.0–40.0%)	44.0% (28.8–60.0%)	18.0% (10–18.8%)
Periprocedural complication	2.9% (1/35)	0.0% (0/16)	6.3% (1/16)	0.0% (0/8)
Restenosis	25.8% (9/35)	12.5% (2/16)	31.2% (5/16)	25.0% (2/8)
Symptomatic restenosis	2.9% (1/35)	0.0% (0/16)	0.0% (0/16)	25.0% (2/8)
Asymptomatic restenosis	20.0% (7/35)	12.5% (2/16)	31.2% (5/16)	0.0% (0/8)
**Wall enhancement change**				
weakened	53.6% (15/28)	75.0% (9/12)	36.4% (4/11)	40.0% (2/5)
constant	42.9% (12/28)	25.0% (3/12)	63.7% (7/11)	40.0% (2/5)
obvious	3.6% (1/28)	0.0% (0/12)	0.0% (0/11)	20.0% (1/5)

### Image Quality

**Figure 2:**
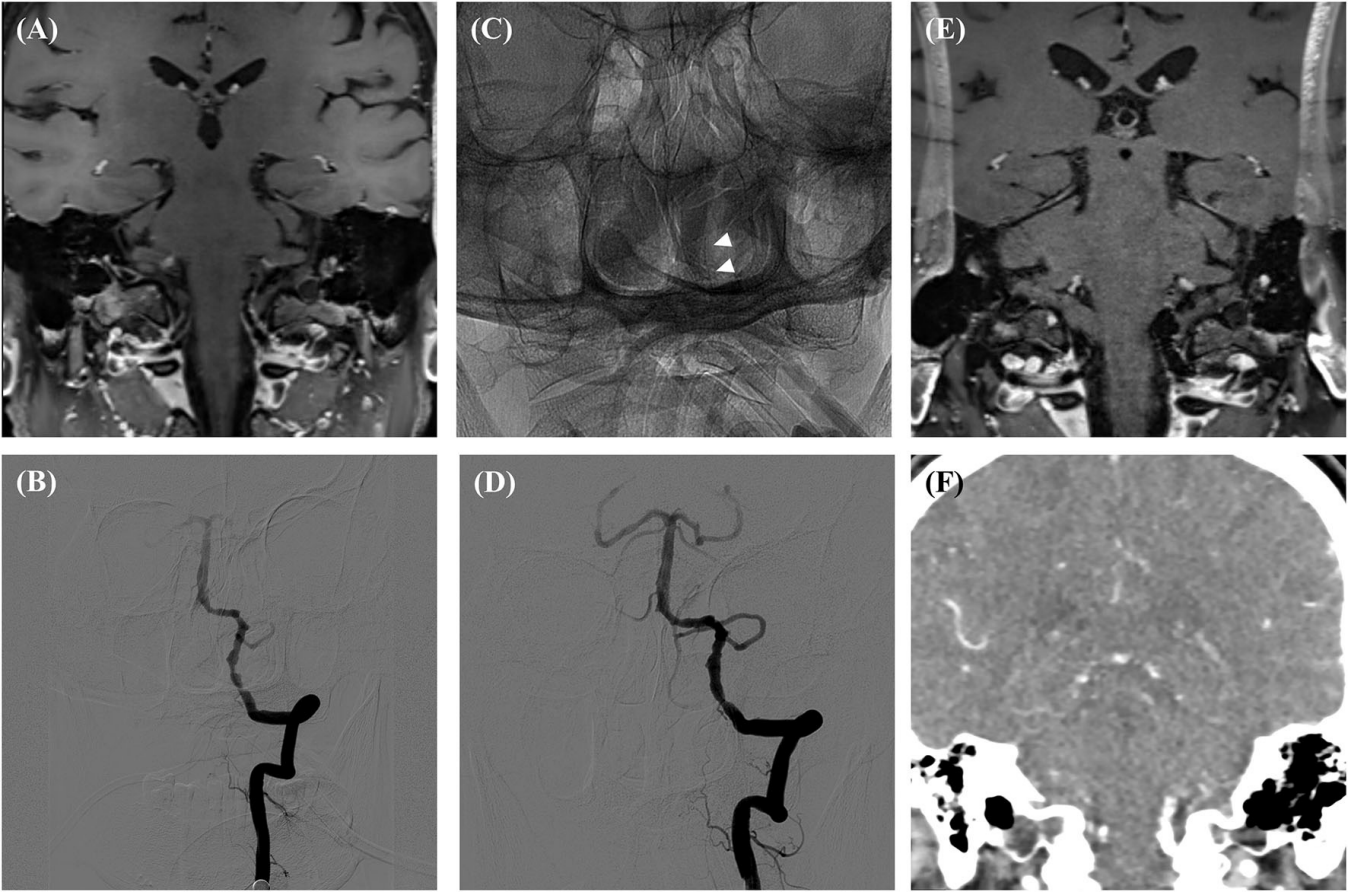
Preprocedural HR-MR **(A)**, Preprocedural DSA **(B)**, DSA immediately after the procedure **(C,D)**, follow-up HR-MR **(E)** and CTA **(F)**. There was a signal drop at the level of the vessel in the follow-up HR-MR images. White arrows showed the Apollo stent.

The overall image quality for HR-MR was 2.8 ± 0.4 (1 point in 1 case, 2 points in 5 cases, and 3 points in 34 cases). In one case with a stainless-steel stent (Apollo; MicroPort Medical, Shanghai, China), there was a signal drop at the level of the vessel, which did not allow evaluation of the vessel lumen (Figure 2).

### Inter-Reader Agreement of HR-MR and TOF-MR

**Figure 3:**
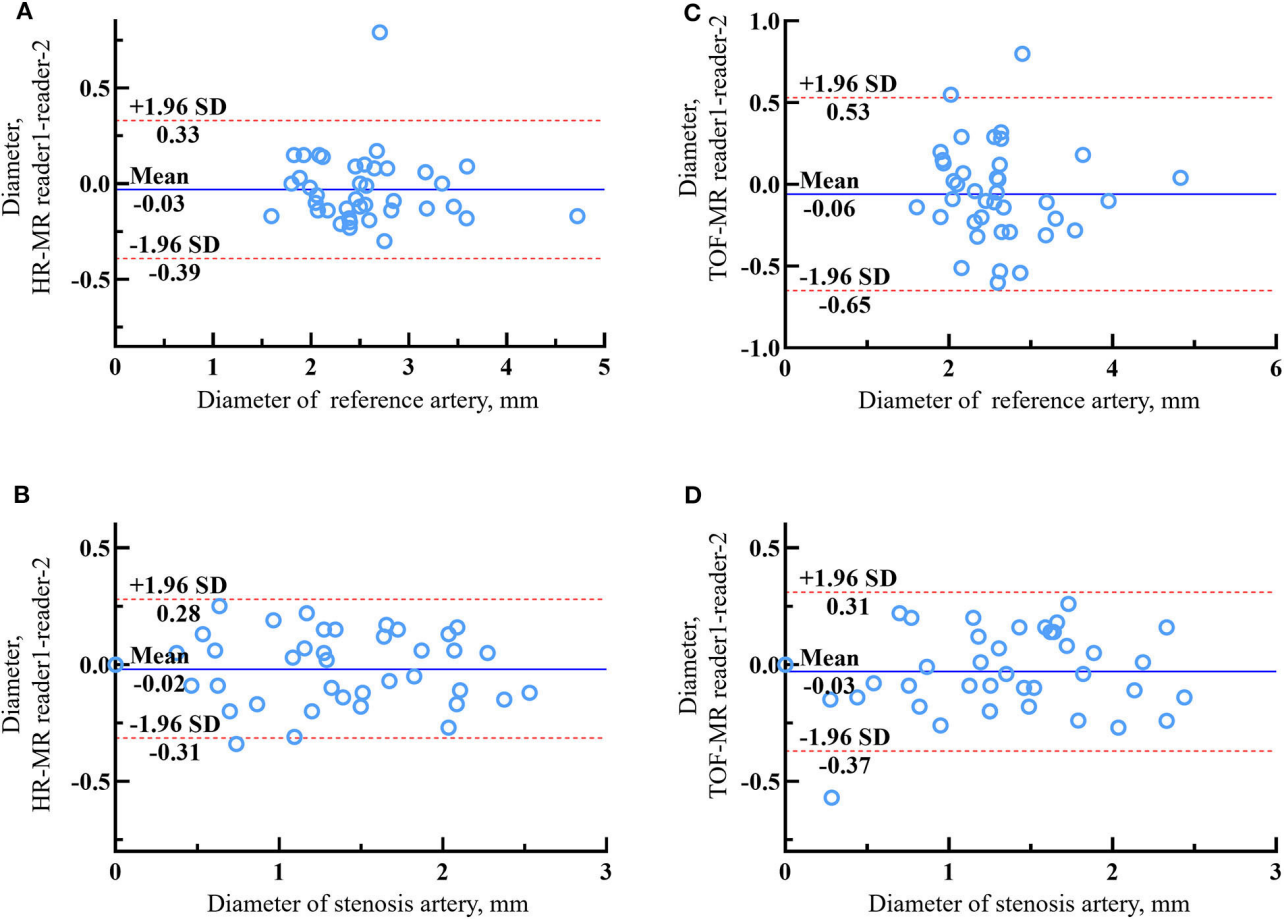
The Bland–Altman plots of lumen diameter measurements between reader 1 and reader 2 of reference artery and stenosis artery on HR-MR images **(A,B)** and tetralogy of fallot (TOF)-MR images **(C,D)**. The solid lines represent the mean difference and the dashed lines indicate the 95% limits of agreement.

In 39 lesions with sufficient image quality, two observers measured the diameter of the stenosis and reference vessel. The ICCs of the HR-MR group were 0.956 and 0.973, and the ICCs of the TOF–MRI group were 0.890 and 0.962, respectively. The Bland–Altman plots of the diameter of the stenosis and reference vessel between reader 1 and reader 2 are shown in Figure 3. The mean differences were −0.015 and −0.027 mm in the HR-MRI group and −0.034 and −0.061 mm in the TOF–MR group (Table 2).

**Table 2:** Inter-reader agreement of arteries without total occlusion on time-of-flight magnetic resonance (TOF-MR) and HR-MR images.

**Measurement methods**	**ICC**	***P*** **-value**	**Reader 1**	**Reader 2**	**Mean difference ± SE**	**Limits of agreement**
**TOF MR**						
Reference diameter	0.890	*P <* 0.001	2.574 ± 0.627	2.635 ± 0.653	−0.061 ± 0.047	−0.646, 0.525
Stenosis diameter	0.962	*P <* 0.001	1.290 ± 0.650	1.324 ± 0.626	−0.034 ± 0.027	−0.375, 0.367
**HR MR**						
Reference diameter	0.956	*P <* 0.001	2.563 ± 0.608	2.590 ± 0.621	−0.027 ± 0.028	−0.386, 0.332
Stenosis diameter	0.973	*P <* 0.001	1.320 ± 0.650	1.336 ± 0.657	−0.015 ± 0.024	−0.314, 0.283

*Data are presented as mean eaSD. Numbers in parentheses are 95% CI.*

*ICC, intraclass correlation coefficient*.

### Agreement Between HR-MR TOF-MR and DSA

**Figure 4:**
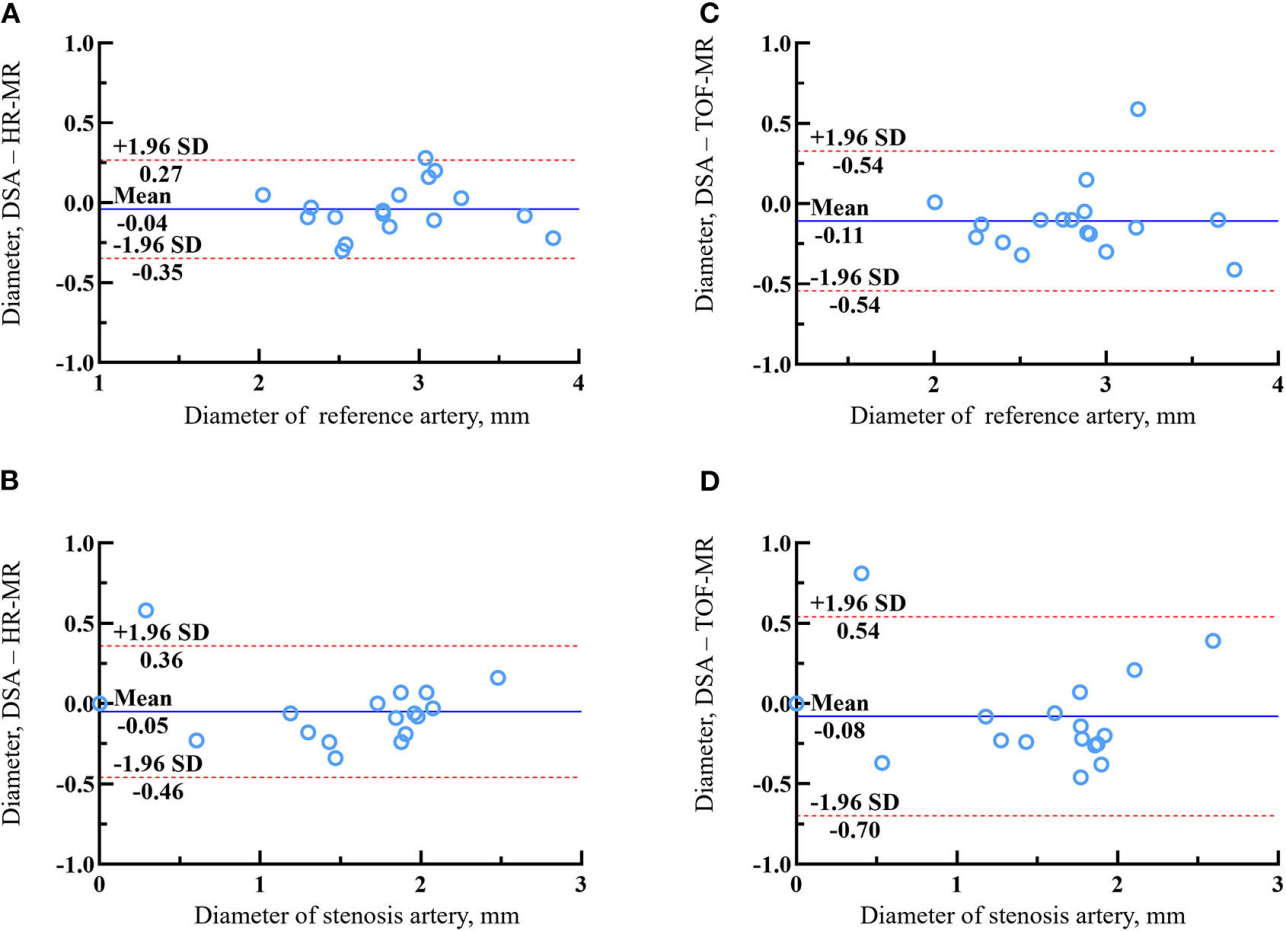
The Bland–Altman plots of lumen diameter measurements of reference artery and stenosis artery between HR-MR images **(A,B)** or TOF-MR images **(C,D)** and DSA. The solid lines represent the mean difference and the dashed lines indicate the 95% limits of agreement.

Of the 40 arteries, 16 arteries were followed-up by DSA. The agreements in the quantitative measurements between HR-MR and DSA and between TOF-MR and DSA are given in Table 3. The Spearman correlation coefficient indicated excellent agreement for all metrics (all the coefficients ≥0.90) between HR-MR and DSA. The Bland–Altman plots of the vessel diameter between HR-MR and between DSA and TOF-MR are presented in Figure 4. In the HR-MR group, the absolute (relative) difference of stenosis diameter and absolute (relative) difference of reference diameter were −0.051 (3.3%) mm and −0.040 (1.4%) mm, respectively. In addition, the values were −0.083 mm (5.3%) and −0.108 mm (3.8%) in the TOF-MR group, respectively (Table 4).

**Table 3:** Correlation coefficient between digital subtraction angiography (DSA) and HR-MR in the different measurement metrics.

**Measurement metrics**	**Spearman's correlation coefficient**
**Pre-procedural**	
Luminal diameter of reference vessel	0.924
Luminal diameter of stenotic lesion	0.964
Degree of stenosis	0.936
**Follow-up**	
Luminal diameter of reference vessel	0.988
Luminal diameter of stenotic lesion	0.960
Degree of stenosis	0.809

**Table 4:** Summary of agreement between TOF-MR or HR-MR images and DSA.

**Measurement methods**	**ICC**	***P*** **-value**	**Reader 1**	**Reader 2**	**Mean difference ± SE**	**Limits of agreement**
**TOF MR**						
Reference diameter	0.890	*P <* 0.001	2.574 ± 0.627	2.635 ± 0.653	−0.061 ± 0.047	−0.646,0.525
Stenosis diameter	0.962	*P <* 0.001	1.290 ± 0.650	1.324 ± 0.626	−0.034 ± 0.027	−0.375,0.367
**HR MR**						
Reference diameter	0.956	*P <* 0.001	2.563 ± 0.608	2.590 ± 0.621	−0.027 ± 0.028	−0.386,0.332
Stenosis diameter	0.973	*P <* 0.001	1.320 ± 0.650	1.336 ± 0.657	−0.015 ± 0.024	−0.314,0.283

*Data are presented as mean eaSD. Numbers in parentheses are 95% CI.*

*ICC, intraclass correlation coefficient*.

### Diagnostic Accuracy of HR-MR Imaging

Using DSA as the reference, the sensitivity; specificity; positive predictive value; negative predictive value; and accuracy of HR-MR for detecting stenosis at >50, >70%, and restenosis are presented in Table 5. HR-MR had a high level of sensitivity and specificity for detecting stenosis >50%, stenosis >70%, and total occlusion.

**Table 5:** Prediction performance of HR-MR in different stenosis degrees.

**Stenosis degree**	**SEN**	**SPN**	**PPV**	**PNV**	**ACC**
Stenosis >50%	1	0.86	0.75	1	0.917
Stenosis >70%	1	1	1	1	1
Restenosis	1	0.86	0.75	1	0.917

*SEN, SPN, PPV, PNV, and ACC are short of sensitivity, specificity, positive predictive value, negative predictive value, and accuracy*.

### Wall Enhancement During Follow-Up

Of the 28 stenotic lesions with both the preprocedural and follow-up HR-MR images, wall enhancement was seen in 22 (78.6%) at baseline. During follow-up, the enhancement weakened in 15 lesions and remained constant in 12, but was clear in 1 case. Compared with the PTA subgroup, more lesions weakened in the DCB subgroup [75.0 (9/12) vs. 36.4% (4/11), *p* = 0.06].

## Discussion

This study found that HR-MR had good accuracy for the assessment of intracranial reference vessels and lesion vessels in the follow-up of patients with ICAD after EVT. The measurement results of HR-MR were highly consistent with DSA. Accurate lumen information can be provided even after most stents are placed. In addition, HR-MR can also provide tube wall images, which is helpful to further evaluate the postoperative situation. Therefore, HR-MR may be a useful imaging option comparable to DSA for the evaluation of stenosis and the detection of restenosis after EVT.

In this study, we evaluated the consistency of HR-MR between the two readers. Our results showed excellent interobserver reproducibility for the measurement of the lumen diameter of stenosis and reference vessels (ICC > 0.9). At the same time, HR-MR showed good agreement (ICC > 0.9) and significant correlations (Spearman *R* >0.8) with DSA regarding the degree of stenosis and the minimal luminal diameter. Using DSA as a reference, HR-MR performed a high level of sensitivity and specificity at diagnostic of stenosis. Compared with TOF-MR, HR-MR seems to have higher interobserver reproducibility and agreement with DSA. This may be because HR-MR can provide better resolution and visualize the boundaries of the vessel wall that can help readers distinguish the vascular boundaries.

High-resolution magnetic resonance may be a useful imaging method regarding the diagnosis and might also help to compensate for the limitations of luminal angiography due to the additional information beyond the luminal characterization. Atherosclerotic plaque enhancement may be associated with neovessel formation, active inflammatory cells, and fibrous cap thinning (18, 19). Several studies have reported that enhancing atherosclerotic plaque is more common in patients with ischemic symptoms or positive DWI findings, suggesting its instability and vulnerability (20–22). Antiproliferative drugs in DCBs can inhibit smooth muscle cell proliferation due to the natural immune inflammatory response (23). Recent data have shown some convincing results in reducing the restenosis rate after EVT in patients with ICAD (24–28). This study demonstrated a higher proportion of plaque enhancement weakening during follow-up in the DCB group. This may be related to the low rate of restenosis.

High-resolution magnetic resonance may have some impact on clinical practice. It may characterize the nature of the occlusion lesion and distinguish different etiologies. Furthermore, it can trace the vessel course and calibrate the distal lumen even in areas of stasis flow. This may be helpful in the therapeutic planning, guiding the selection of balloon or stent size at the occlusive segment that is invisible on DSA. HR-MR can also be used as an alternative follow-up method after EVT of ICAD due to its high accuracy for restenosis and non-radioactivity. For restenosis cases, the characteristics of the vessel wall may guide us to choose a comprehensive treatment (including lipid-lowering, anti-inflammatory, and DCB) rather than just angioplasty.

There are several limitations in this study. First, a retrospective study with a small number of cases may have resulted in limitations in selection bias and statistical power. The accuracy of HR-MR has yet to be confirmed by data from larger series. Second, not all follow-up images were 3D. In 2D images, the eccentricity of the stenotic lesion may have biased the lumen diameter measurement. Third, almost all the cases in this study were atherosclerotic lesions. The conclusions drawn from this series of cases are not necessarily applicable to cases of other causes, such as dissection. The imbalance of analyzed arterial segments may have led to selection bias. However, stratified analysis was not available due to the small sample size. Despite these limitations, we believe that our study reflects exploration in actual clinical practice. We hope that further research can be extended to larger samples and other diverse etiologies and apply HR-MR to the clinical prognosis field.

## Conclusion

High-resolution magnetic resonance can provide high-resolution images of both the vessel wall and lumen. It could be a reliable non-invasive method for demonstrating the vessel lumen and diagnostic follow-up after endovascular recanalization for ICAD.

## Data Availability Statement

The original contributions presented in the study are included in the article/supplementary material, further inquiries can be directed to the corresponding author.

## Ethics Statement

The studies involving human participants were reviewed and approved by the Institutional Review Board of Beijing Hospital. The patients/participants provided their written informed consent to participate in this study.

## Author Contributions

JW and SZ reviewed the image data. All authors contributed to the article and approved the submitted version.

## Funding

This study was funded by the Beijing Hospital Clinical Research 121 Project (BJ-2018-086 and BJ-2018-202), Capital's Funds for Health Improvement and Research (2020-4-4053).

## Conflict of Interest

The authors declare that the research was conducted in the absence of any commercial or financial relationships that could be construed as a potential conflict of interest.

## Publisher's Note

All claims expressed in this article are solely those of the authors and do not necessarily represent those of their affiliated organizations, or those of the publisher, the editors and the reviewers. Any product that may be evaluated in this article, or claim that may be made by its manufacturer, is not guaranteed or endorsed by the publisher.
